# Surgical Resection of Pulmonary Metastases from Melanoma in Oligometastatic Patients: Results from a Multicentric Study in the Era of Immunoncology and Targeted Therapy

**DOI:** 10.3390/cancers15092462

**Published:** 2023-04-25

**Authors:** Elisa Meacci, Dania Nachira, Maria Teresa Congedo, Mohsen Ibrahim, Gianluca Pariscenti, Francesco Petrella, Monica Casiraghi, Alessandro De Stefani, Laura del Regno, Ketty Peris, Elizabeth Katherine Anna Triumbari, Giovanni Schinzari, Ernesto Rossi, Leonardo Petracca-Ciavarella, Maria Letizia Vita, Marco Chiappetta, Alessandra Siciliani, Valentina Peritore, Mattia Manitto, Lucia Morelli, Edoardo Zanfrini, Diomira Tabacco, Giuseppe Calabrese, Claudia Bardoni, Jessica Evangelista, Lorenzo Spaggiari, Stefano Margaritora

**Affiliations:** 1 Department of General Thoracic Surgery, Fondazione Policlinico Universitario “A. Gemelli”, Istituto di Ricovero e Cura a Carattere Scientifico (IRCCS), Università Cattolica del Sacro Cuore, 00168 Rome, Italy; 2 Thoracic Surgery Unit, Sant’Andrea Hospital, University of Rome La Sapienza, 00185 Rome, Italy; 3 IRCCS Ospedale Policlinico San Martino, 16132 Genova, Italy; 4 Department of Thoracic Surgery, IEO, European Institute of Oncology IRCCS, 20141 Milan, Italy; 5 Department of Oncology and Hemato-Oncology, University of Milan, 20122 Milan, Italy; 6 Dermatology Unit, Fondazione Policlinico Universitario A. Gemelli IRCCS, Università Cattolica del Sacro Cuore, 00168 Rome, Italy; 7 Nuclear Medicine Unit, G-STeP Radiopharmacy Research Core Facility, Department of Radiology, Radiotherapy and Hematology, Fondazione Policlinico Universitario A. Gemelli IRCCS, 00168 Rome, Italy; 8 Medical Oncology Unit, Fondazione Policlinico Universitario A. Gemelli IRCCS, Università Cattolica del Sacro Cuore, 00168 Rome, Italy; 9 Service de Chirurgie Thoracique et de Trasplantation Pulmonaire, Hôpital Européen Georges Pompidou, 75015 Paris, France

**Keywords:** lung metastases, malignant melanoma, melanoma, pulmonary metastasectomy, metastatic melanoma

## Abstract

**Simple Summary:**

Since the introduction of effective systemic therapies (ESTs) (in the form of both targeted and immuno-based therapies) in the treatment of malignant melanoma, the prognosis of metastatic patients has dramatically changed. The role of metastasectomy in oligometastatic patients has rarely been addressed in this new pharmacological era. Even though lungs represent the most frequent site of melanoma metastases, only limited data are available on the role of surgery in isolated pulmonary metastases from malignant melanoma (PmMM). In this study, we describe the outcomes of patients who underwent metastasectomy of PmMM in the era of ESTs, and identified prognostic factors affecting survival in our multicentric experience. To the best of our knowledge, this is the first paper analyzing in detail the impact of metastasectomy of PmMM in the era of ESTs.

**Abstract:**

In the last decade, the emergence of effective systemic therapies (ESTs) in the form of both targeted and immuno-based therapies has revolutionized the treatment of patients with advanced stage III and stage IV melanoma. Even though lungs represent the most frequent site of melanoma metastases, only limited data are available on the role of surgery in isolated pulmonary metastases from malignant melanoma (PmMM) in the era of ESTs. The aim of this study is to describe the outcomes of patients who underwent metastasectomy of PmMM in the era of ESTs, in order to identify prognostic factors affecting survival and to provide a framework for more informed patient selection of treatmeant with lung surgery in the future. Clinical data of 183 patients who underwent metastasectomy of PmMM between June 2008 and June 2021 were collected among four Italian Thoracic Centers. The main clinical, surgical and oncological variables reviewed were: sex, comorbidities, previous oncological history, melanoma histotypes and primary site, date of primary cancer surgical treatment, melanoma growth phase, Breslow thickness, mutation pattern disease, stage at diagnosis, metastatic sites, DFI (Disease Free Interval), characteristics of lung metastases (number, side, dimension, type of resection), adjuvant therapy after lung metastasectomy, site of recurrence, disease-free survival (DFS) and cancer-specific survival (CSS; defined as the time interval between the first melanoma resection or lung metastasectomy and death from cancer). All patients underwent surgical resection of the primary melanoma before lung metastasectomy. Twenty-six (14.2%) patients already had a synchronous lung metastasis at the time of primary melanoma diagnosis. A wedge resection was performed in 95.6% of cases to radically remove the pulmonary localizations, while an anatomical resection was necessary in the remaining cases. The incidence of major post-operative complications was null, while only 21 patients (11.5%) developed minor complications (mainly air leakage followed by atrial fibrillation). The mean in-hospital stay was 4.46 ± 2.8 days. Thirty- and sixty-day mortality were null. After lung surgery, 89.6% of the population underwent adjuvant treatments (47.0% immunotherapy, 42.6% targeted therapy). During a mean FUP of 107.2 ± 82.3 months, 69 (37.7%) patients died from melanoma disease, 11 (6.0%) from other causes. Seventy-three patients (39.9%) developed a recurrence of disease. Twenty-four (13.1%) patients developed extrapulmonary metastases after pulmonary metastasectomy. The CSS from melanoma resection was: 85% at 5 years, 71% at 10 years, 54% at 15 years, 42% at 20 years and 2% at 25 years. The 5- and 10-year CSS from lung metastasectomy were 71% and 26%, respectively. Prognostic factors negatively affecting CSS from lung metastasectomy at multivariable analysis were: melanoma vertical growth (*p* = 0.018), previous metastatic sites other than lung (*p* < 0.001) and DFI < 24 months (*p* = 0.007). Our results support the evidence that surgical indication confirms its important role in stage IV melanoma with resectable pulmonary metastases, and selected patients can still benefit from pulmonary metastasectomy in terms of overall cancer specific survival. Furthermore, the novel systemic therapies may contribute to prolonged survival after systemic recurrence following pulmonary metastasectomy. Patients with long DFI, radial growth melanoma phase and no site of metastatization other than lung seem to be the best candidate cases for lung metastasectomy; however, to drive stronger conclusions, further studies evaluating the role of metastasectomy in patients with iPmMM are needed.

## 1. Introduction

Malignant melanoma (MM) represents the most aggressive form of skin cancer. It is responsible for 0.6% of deaths owing to cancers of all sites and 65% of deaths for skin cancers [1].

According to 2020 statistics, the number of new MM cases in the United States was 100,350 with 6850 deaths, and the proportion is expected to steadily increase because of the accumulated high levels of exposure to ultraviolet light of the ageing population [2]. 

The prognosis of MM is excellent when detected at early-stage (lesions not thicker than 1.0 mm have five-year survival rates greater than 90% [1]), but it becomes extremely poor in advanced stages. 

About 4% of all MM show distant metastases at diagnosis [3], with the most common site of metastases represented by the lungs, affecting up to 30% of metastatic patients [4].

The treatment of metastatic melanoma has historically been challenging as a result of the ineffectiveness of traditional chemotherapy [5]. 

Systemic treatments based on traditional cytotoxic chemotherapeutic agents (i.e., dacarbazine, temozolomide, paclitaxel, DTIC, cisplatin, BCNU, tamoxifen and cytokines such as IL-2 and interferon) have failed to control advanced disease, with response rates ranging from 15 to 20%, progression-free survival of 4.8 months and survival of 9.0 months [3,6,7].

In this daunting landscape, the encouraging results achieved after metastasectomy in selected surgically treated oligometastatic patients with stage IV colorectal and renal cancer [8,9] led to surgical resection being considered the only viable option in the era of cytotoxic chemotherapeutic agents. Indeed, it became an integral part of the treatment of oligometastatic melanoma [10]. In retrospective series of such patients, surgery was consistently associated with improved survival outcomes compared to patients managed non-operatively or to those operated on with palliative intent [11].

The South West Oncology Group (SWOG) Clinical Trial 9430 reported a four-year overall survival (OS) of 31% in stage IV melanoma oligometastatic patients with completely resected disease [12]. Similar data derived from the Multicentre Selective Lymphadenectomy Trial (MSLT)-I. Here, patients chosen for surgery as a component of treatment had an improved four-year OS of 21% vs. a 7% OS for patients treated with systemic treatment alone [13].

In selected patients with isolated pulmonary metastases from malignant melanoma (PmMM), a median survival of up to 18.3 months and five-year survival rates of up to 35.1% have been described after surgical resection [14], endorsing the role of lung metastasectomy in metastatic melanoma as a complementary treatment with double intent: palliation, with symptom resolution in the majority of patients [15], and curative intent in patients with limited volume metastases, where a complete resection of metastases could be achieved.

In the last decade, the emergence of effective systemic therapies (ESTs) in the form of both targeted and immuno-based therapies, including BRAF- and MEK-inhibitors in association with programmed cell death (PD-1) inhibitors and cytotoxic T-lymphocyte-associated protein 4 (CTLA-4), has revolutionized the treatment of patients with advanced stage III and stage IV melanoma [16,17,18], offering a four-year OS of up to 53%. However, the median progression-free survival, even in a polypharmacological setting, was 11.5 months, meaning that approximately 50% of patients would have experienced recurrence within one year [19].

Although the value of curative surgery in oligometastatic melanoma in the era of ESTs has not yet been validated in phase III prospective studies, data from phase II studies are available [12]. Their data suggest that surgical removal of distant metastases should be considered as a therapeutic option in selected patients, offering potential for long-term disease control [III, C] [20]. A phase III randomized trial evaluating the efficacy of an allogenic melanoma vaccine reported a notable 43% five-year OS in patients with stage IV melanoma following complete resection of up to five metastatic sites [21]. 

Even though lungs represent the most frequent site of melanoma metastases, only limited data are available on the role of surgery in PmMM in the era of ESTs.

The aim of this study is to describe the outcomes of patients who underwent metastasectomy of PmMM in the era of ESTs, in order to identify prognostic factors affecting survival and to provide a framework for more informed patient selection of treatment with lung surgery in the future.

To the best of our knowledge, this is the first paper analyzing the impact of metastasectomy of PmMM in the era of immune and targeted therapies.

## 2. Methods

Clinical data of 183 oligometastatic patients who underwent metastasectomy of PmMM between June 2008 and June 2021 were collected among four Italian Thoracic Centers.

The study was approved by our IRB, and therefore performed in accordance with the ethical standards of the Declaration of Helsinki and its later amendments. Individual informed consent was waived due to the retrospective nature of the study and the anonymity of patients enrolled.

The main clinical, surgical and oncological variables reviewed were: sex, comorbidities, previous oncological history, melanoma histotypes and primary site, date of primary cancer surgical treatment, melanoma growth phase (vertical or radial), Breslow thickness, mutation pattern (BRAF, RAS, c-Kit, N-RAS), neurovascular invasion and neurotropism, disease stage at diagnosis, type of adjuvant therapy (immunotherapy, targeted therapy, chemotherapy), metastatic sites, date of lung metastasectomy, disease-free interval (DFI; time interval between melanoma removal and first pulmonary metastasis diagnosis), characteristics of lung metastases (number, side, dimension, type of resection), date of last follow-up (FUP), status at FUP, adjuvant therapy after lung metastasectomy, site of recurrence, disease-free survival (DFS; time interval between lung metastasectomy and date of disease recurrence) and cancer specific survival (CSS; defined as the time interval between the first melanoma resection or lung metastasectomy and death for cancer). The main immunotherapies adopted after melanoma removal were: ipilimumab, nivolumab, pemprolizumab; targeted therapies were: dabrafenib+trametinib, vemurafenib+cobimetinib. 

Each clinical case had been discussed by internal multidisciplinary teams (involving radiologists, oncologists, dermatologists and thoracic surgeons) for the approval of the indication for lung metastasectomy. Indications for complete pulmonary metastasectomy included radical resection of all visible tumor lesions in patients with only lung metastases. Patients who had undergone interventional diagnostic procedures were excluded. In all patients, the primary melanoma was under control.

Before lung metastasectomy, all patients were evaluated by routine blood tests, electrocardiography (or other cardiologic second level tests, if necessary), pulmonary function test and total-body computed tomography (CT). Lung resection was performed under general anesthesia and double-lumen intubation. A wedge or major anatomic resection (in case of central or large lesions) with macroscopic tumor-free borders was performed by thoracotomy or Video-Assisted Thoracic Surgery (VATS), according to the preferred surgical approach of each center.

### Statistical Analysis

All categorical variables were reported as absolute numbers and percentages (%), continuous variables as mean and standard deviation. Kolmogorov–Smirnov test was used to evaluate normal distribution of data. Categorical variables were compared by Chi-squared test and continuous variables by independent sample Student’s *t*-test if normal distributed, or by Mann–Whitney U-test if not normal.

Survival and disease-free analyses were performed by Kaplan–Meier method; Log-Rank test was used to assess differences in survival. Univariate analysis with a Cox proportional hazard model was conducted to evaluate prognostic factors. All covariates with *p*-value < 0.1 at univariate analysis were selected for Multivariable Cox regression analysis to assess factors independently affecting survival. A *p*-value < 0.05 was considered statistically significant. 

Statistical analysis was performed using IBM SPSS Statistics for Macintosh (version 25.0, IBM Corp, Armonk, NY, USA). 

## 3. Results

The main clinical characteristics of the 183 analyzed patients are reported in Table 1. 

All patients underwent surgical resection of the primary melanoma before lung metastasectomy.

The main melanoma site was skin in 172 cases (94.0%), and the predominant histology in the series was superficial spreading (95 cases, 51.9%) followed by nodular type (65 cases, 35.5%). In 26.2% of cases (48 patients), melanoma was diagnosed at IIA stage, followed by IB stage in 19.7% of cases (36 patients) (Table 1). Sixty-four patients (34.9%) had a proven BRAF mutation. Seventy-five (41.0%) and 18 (9.8%) patients underwent adjuvant immunotherapy and targeted therapy after melanoma removal, respectively (Table 1).

Twenty-six (14.2%) patients already had a synchronous lung metastasis at the time of primary melanoma diagnosis. The mean DFI was 62.93 ± 61.19 months, and the mean diameter of lung lesions was 12.4 ± 5.6 mm. Twenty-one (11.5%) patients developed multiple lung metastases. A wedge resection was performed in 95.6% of cases to radically remove the pulmonary localizations, while an anatomical resection was necessary in the remaining cases. The incidence of major post-operative complications was null, while only 21 patients (11.5%) developed minor complications (mainly air-leakage followed by atrial fibrillation). The mean in-hospital stay was 4.46 ± 2.8 days. Thirty- and sixty-day mortality were null.

After lung surgery, 89.6% of the population underwent adjuvant treatments (47.0% immunotherapy, 42.6% targeted therapy), Table 1. 

During a mean FUP of 107.2 ± 82.3 months, 69 (37.7%) patients died from melanoma disease, and 11 (6.0%) from other causes. Seventy-three patients (39.9%) developed a recurrence of disease. Twenty-four (13.1%) patients developed extrapulmonary metastases after pulmonary metastasectomy.

**Figure 1:**
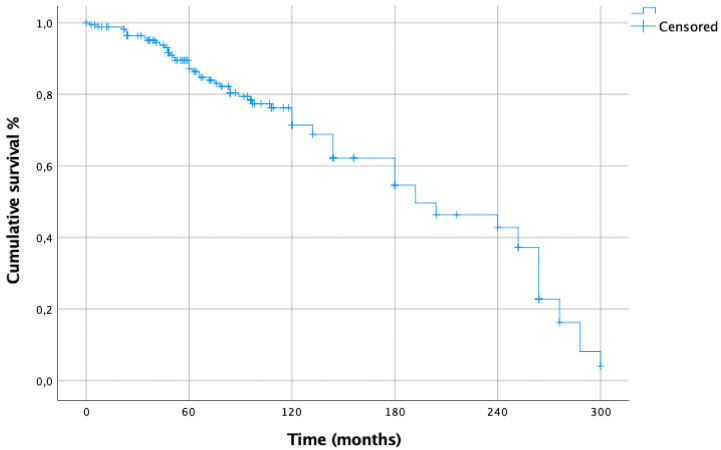
CSS from primary melanoma resection.

**Figure 2:**
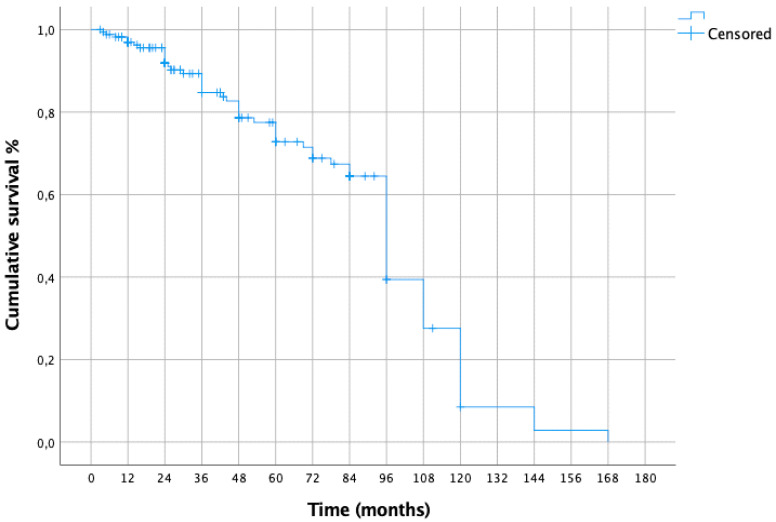
CSS from metastasectomy.

The CSS from melanoma resection was: 85% at 5 years, 71% at 10 years, 54% at 15 years, 42% at 20 years and 2% at 25 years (Figure 1). The 5- and 10-year CSS from lung metastasectomy were 71% and 26%, respectively (Figure 2).

**Figure 3:**
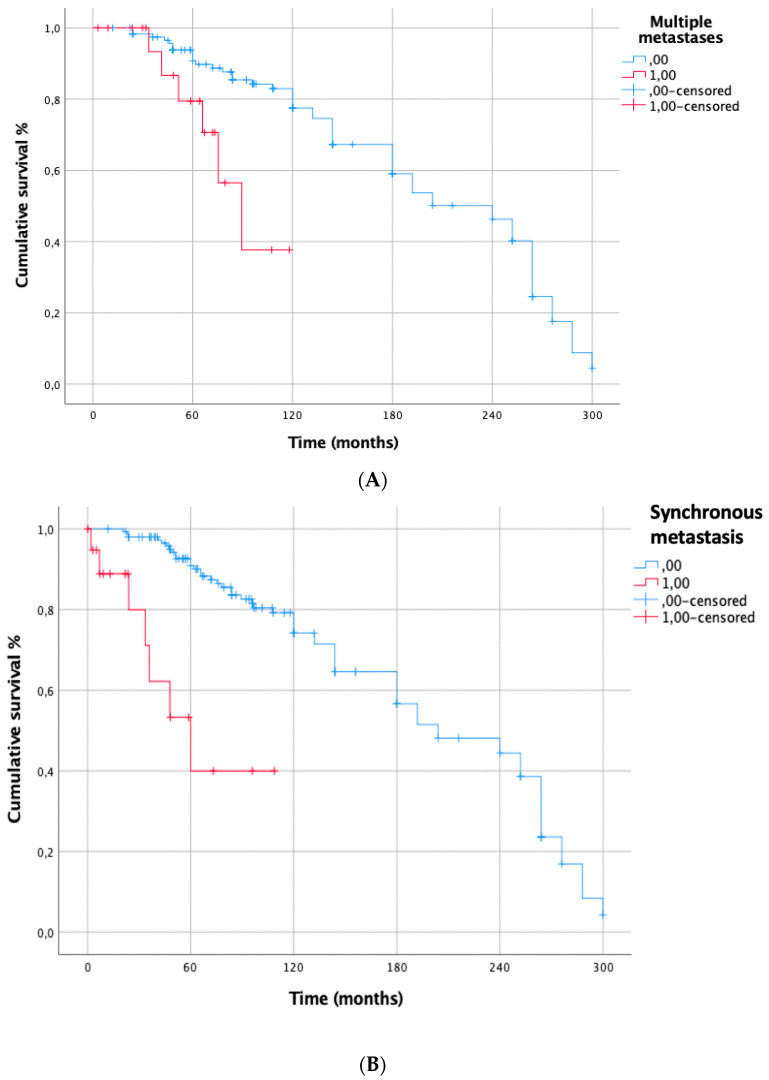
(**A**): CSS from primary melanoma for multiple metastases. Single metastasis: 5-y: 90%, 10-y: 77%; 15-y: 58%; 20-y: 45% multiple metastases: 5-y: 71%; 10-y: 39%; *p* = 0.002. (**B**): CSS from primary melanoma for synchronous metastases. Metacronous metastasis: 5-y: 88%; 10-y: 74%; 15-y: 56%; 20-y: 44%, synchronous metastasis: 5-y: 52%; 10-y: 39%, *p* < 0.001.

**Figure 4:**
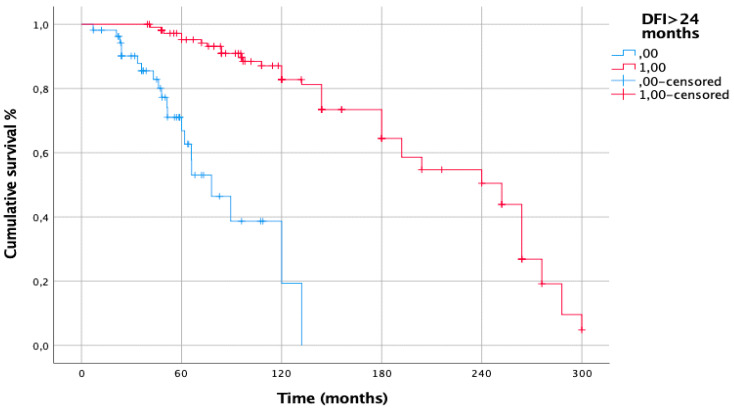
CSS from primary melanoma for DFI > 24 months. DFI > 24 months: 5-y: 95%; 10-y: 82%; 15-y: 63%; 20-y: 50%; DFI ≤ 24 months: 5-y: 52%; 10-y: 19%; *p* < 0.001.

The prognostic factors negatively affecting CSS from primary melanoma resection at univariate analysis were: multiple lung metastases (*p* = 0.002), synchronous lung metastases (*p* < 0.001) (Figure 3A,B), previous metastatic sites other than lung (*p* = 0.0002); DFI < 24 months (*p* = 0.002). At multivariable Cox regression analysis, only DFI < 24 months (HR [95% CI]: 7.813 [2.787–21.902], *p* < 0.001) (Table 2) confirmed to be an independent prognostic risk factor (Figure 4).

**Figure 5:**
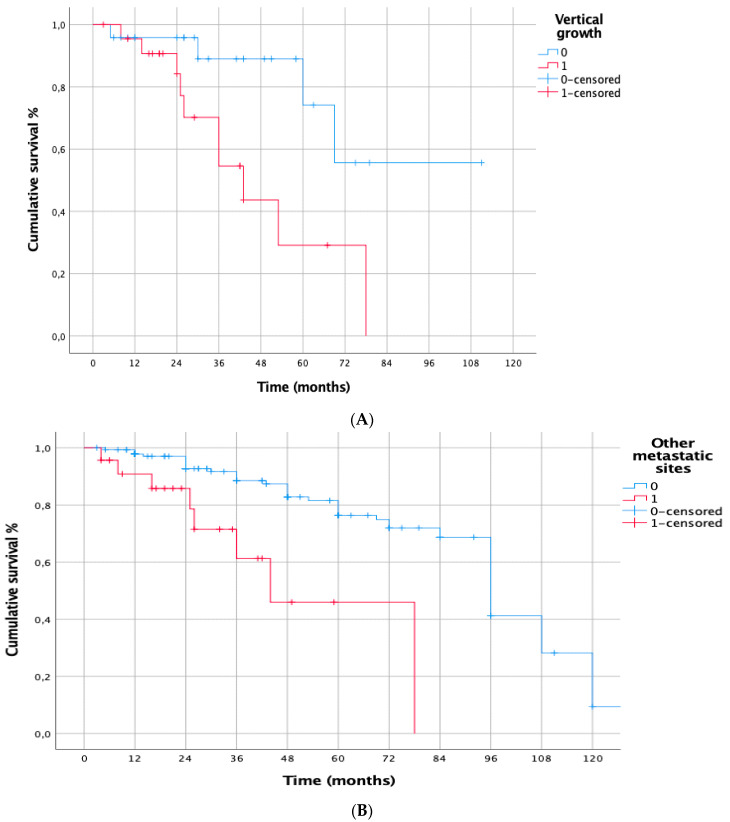

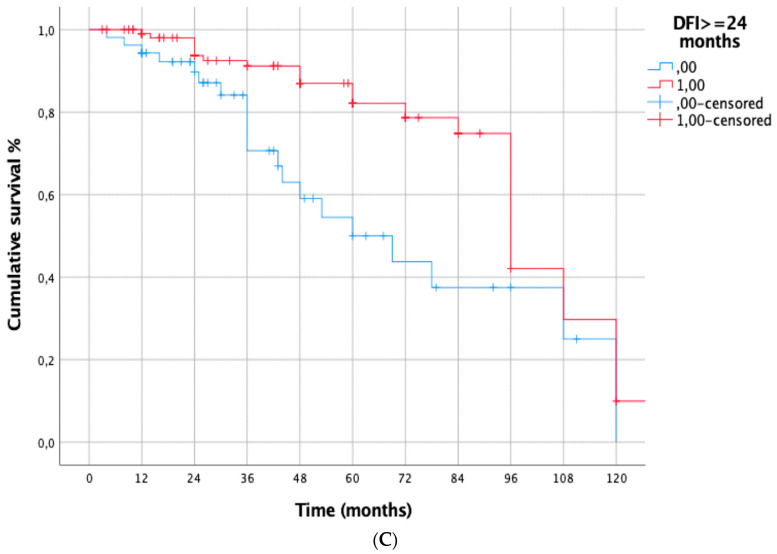
(**A**): CSS from pulmonary metastasectomy for vertical growth. Vertical growth: 2y: 69%; 5y: 28%; radial growth: 2y:89%; 5y: 89%; 8y: 57%; ***p* = 0.022.** (**B**): CSS for other metastatic sites. No previous other metastatic sites: 2y: 91%; 5y: 74%; 10y: 1%; Other previous metastatic sites: 2y: 69%; 5y: 46%; ***p* < 0.01**. (**C**): CSS from pulmonary metastasectomy for DFI >= 24 months. DFI >= 24 months: 2y: 92%; 5y: 81%; 10y: 29%; DFI < 24 months: 2y: 84%; 5y: 44%; 10y: 22%; ***p* = 0.003**.

Prognostic factors negatively affecting CSS from lung metastasectomy were: melanoma vertical growth (*p* = 0.018), previous metastatic sites other than lung (*p* < 0.001) and DFI < 24 months (*p* = 0.007). Melanoma vertical growth (*p* = 0.021) and previous metastatic sites other than lung (*p* = 0.009) confirmed their prognostic role at multivariable analysis (Table 3) (Figure 5A–C).

**Figure 6:**
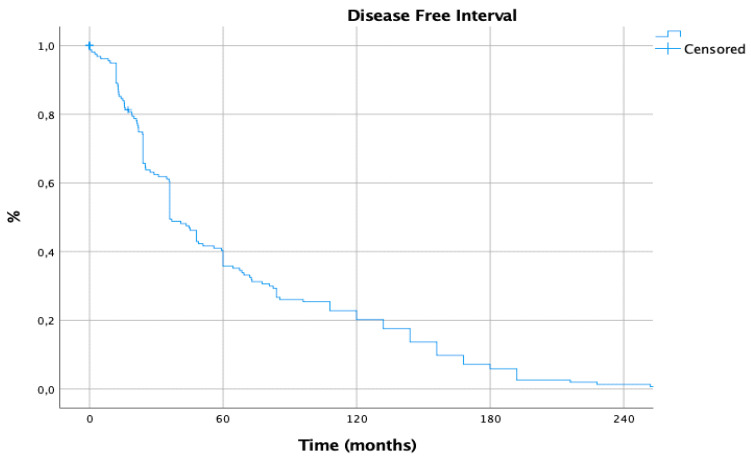
Disease-free interval.

The 5-, 10-, 15- and 20-year DFI between primary cancer and first pulmonary metastasis were 33%, 20%, 6% and 1%, respectively (Figure 6).

Investigating the main variables affecting DFI, only BRAF mutation (*p* = 0.029) resulted in a significant risk factor at univariate analysis, but did not confirm its value at multivariable analysis (Table 4).

**Figure 7:**
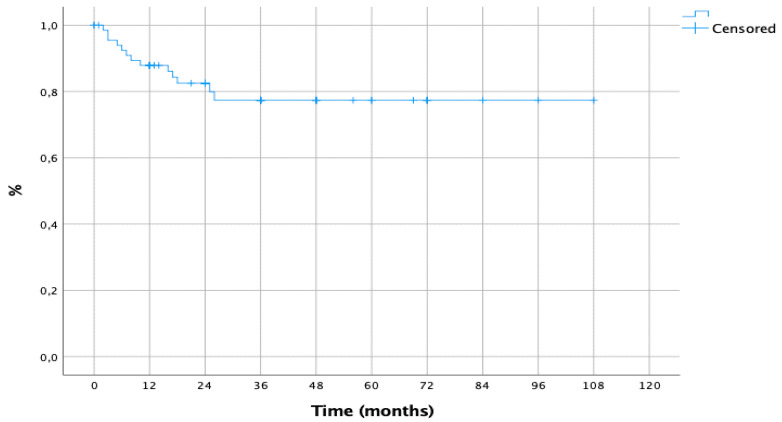
Disease-Free Survival.

Lastly, 1-, 5-and 10-year DFS from pulmonary metastasectomy and first recurrence was 83%, 79% and 79%, respectively (Figure 7). 

The only factor influencing DFS at univariate analysis and confirmed at Cox regression analysis was therapy after metastasectomy (HR [95% CI]: 0.106 [0.028–0.404], *p* = 0.001), as shown in Table 5.

## 4. Discussion

In the last decade, even with the increasing efficacy of ESTs, the use of surgical treatment for metastatic disease has increased across multiple cancer types, including MM, where long term outcomes have benefitted from a dramatic improvement thanks to new therapies. 

Although most studies have focused on the role of metastasectomy in the pre-immunotherapy era so far [8,22,23,24,25,26,27,28,29,30,31,32,33,34,35,36], the increasing number of stage IV MM patients eligible for surgical resection of their metastases makes the evaluation of the role of surgery mandatory in this setting of patients in the era of ESTs.

Even though the lung represents the most common site of metastatization in MM, data on long term outcomes and risk factors affecting survival in patients treated with surgical resection of lung metastases have been rarely evaluated.

This study is the largest analysis of outcomes after lung metastasectomy in patients with PmMM following the introduction of ESTs into the treatment strategy of stage IV MM.

A consistent number of papers have recently focused on the role of Curative Metastasectomy (CM) in patients with abdominal/visceral metastases melanoma. Smith et al., in 2018, evaluated the outcomes of 138 patients undergoing surgery for stage IV melanoma before and after the introduction of new ESTs (2003–2007 vs. 2011–2015), and found trends towards an increase in abdominal metastasectomy, decreased intransit lesion excision and an increase in potentially curative operations for residual oligometastatic disease (15.9% vs. 4.3%, *p* = 0.045), with a significantly prolonged survival after surgery in the after-ESTs cohort (median 16 months vs. 6 months, *p* < 0.001) [37].

The trend described in the case of abdominal metastases has been also reported in the case of PmMM. Hanna et al. in 2018, analyzing data from the Institute for Clinical Evaluative Sciences, observed that prior to first-line dacarbazine (in the years 2007–2009), fewer than 3% of patients affected by PmMM underwent thoracic surgery. With first-line ipilimumab (2010–2015), this percentage rose to 7%. The authors of the study underlined that, for melanoma patients whose first treatment for metastatic disease was thoracic surgery, there was a trend toward better survival in more recent years (log rank *p* = 0.129), with a one-year survival of 72.5% in the period 2007–2009 vs. 85.2% in 2014–2015 [5].

These “paradoxical” results, representing the increased use of surgery with the improvement of systemic therapies, suggest that the more effective systemic disease control offered by the new therapies may endorse the role of surgical resection in metastatic melanoma patients, raising the possibility of performing radical surgery and increasing the number of patients susceptible to surgical resection. 

Lung metastasectomy in patients treated with ESTs seems to dramatically improve long-term outcomes. In a recent review and meta-analysis analyzing outcomes after CM in patients with PmMM, Wankhede et al. [30] observed a five-year OS of 22–40% for patients with lung metastasis who underwent CM, compared with 0–18% for the no-CM patients. The pooled survival outcome in the study supports the curative resection of pulmonary metastasis (HR, 0.48; *p* = 0.002). 

In our study, the CSS from melanoma resection was: 85% at 5 years, 71% at 10 years, 54% at 15 years, 42% at 20 years and 2% at 25 years, while the 5- and 10-year CSS from lung metastasectomy were 71% and 26%, respectively. 

Our results in terms of CSS are impressive if compared with historical series [8,14,22,23,24,25,26,27,28,29,30,31,32,33,34,35,36] published before the introduction of ESTs, where a median OS between 11 and 40 months, and five-year survival rates between 4.5 % and 35.1% were achieved in selected patients with resected pulmonary metastases.

More recently, Hanna et al. [5] evaluated the clinical outcomes after pulmonary metastasectomy in a cohort of 99 cases treated between 2004 and 2012, largely before the advent of new ESTs. The authors found a five-year OS from date of primary diagnosis of 65% and a median OS after first thoracic surgery of 22.9 months, with a median CSS (from first thoracic surgery) of 23.4 months. 

Five-year OS (from first thoracic surgery) and CSS (from first thoracic surgery) were 20.7% and 21.3%, respectively. Therefore, most deaths were cancer-related. Median CSS was slightly longer than median OS, given the small number of non-cancer deaths. The 10-year OS from first thoracic surgery was 16.6%. 

After the introduction of ESTs, Viehof et al. [4] reported the long-term outcomes of 61 consecutive patients treated with complete resection of PmMM between January 2010 and December 2016. At a median follow-up time of 25.6 months after lung metastasectomy, the median OS was 31.3 months, with a two-year survival rate of 54%. 

A remarkable estimated five-year OS rate of 75% was reported by Bello et al. [38] on a cohort of 237 patients with advanced stage III or stage IV melanoma who underwent treatment with ESTs followed by CM. 

The better long-term outcomes found in our cohort may be justified by the a priori selection of patients highly eligible for surgery. In our experience, only 13.1% of patients had previous extrapulmonary metastases vs. 42% in Viehof’s study [4]. The history of extrapulmonary disease at the time of lung metastasectomy negatively influenced the OS following lung metastasectomy (*p* = 0.08) in both Viehof’s series and in ours. Indeed, the presence of previous metastatic sites other than lung negatively influenced CSS from lung metasasectomy at univariate analysis (*p* < 0.001), and confirmed its role as an independent prognostic factor at multivariable analysis (*p* < 0.009). 

Another factor negatively influencing CSS from lung metastasectomy, in our experience, was the vertical growth phase of the primary melanoma. This feature defines the point in lesion evolution when a melanoma acquires the ability to metastasize and kill the patient [39,40,41,42]. 

Vertical growth has already been demonstrated to be strongly associated with nodal metastases development [43] and systemic spreading [44,45] in several previous reports, and has confirmed its negative value in our experience as well.

The meaning of DFI still remains controversial. In our research, a DFI < 24 months (HR [95% CI]: 7.813 [2.787–21.902], *p* < 0.001) represented an independent prognostic risk factor negatively affecting CSS from primary melanoma resection. Similar results have been published in many papers analyzing the outcome of pulmonary metastasectomy [28,29,32]. The major research analyzing the role of DFI was published by Leo et al. [22] on 328 patients from the International Registry of Lung Metastases: patients with a DFI of >36 months had a better long-term survival than patients with a DFI < 36 months (30% vs. 15% at 5 years and 22% vs. 11% at 10 years, *p* < 0.01). A short DFI, however, did not appear to influence OS in the recent experience published by Hanna et al. [5].

The safety of pulmonary metastasectomy is demonstrated by the lack of deaths at 30 days, with no major complications and a small percentage of minor complications (11.5%) in our cohort of PmMM with complete removal of lung metastases. Similar results have been reported in many previous papers [4,5] where, furthermore, median length of in-hospital stay was similar to our findings (4.46 ± 2.8 days). 

The main strength of this paper is that, to the best of our knowledge, this is the largest analysis of outcomes after surgical resection of PmMM in patients operated after the introduction of ESTs and with a long follow-up period (with a mean of 107.2 ± 82.3 months and survival till 20 years).

Of course, it is important to note that there are many intrinsic biases in our study related to its retrospective nature (such as patient selection bias), and there are specific biases that afflict any retrospective multicentric series of metastasectomies. 

The very high CSS reported in our paper may be influenced, as in many other series of metastasectomies, by the high selection of patients showing better chances of long survival who are candidates for surgery. The process of selection itself, rather than the effect of surgery, may be responsible for conferring an apparent survival benefit [46,47,48].

## 5. Conclusions

The introduction of the new ESTs has shown to dramatically improve the outcomes of resectable stage IV MM patients, and represents a rationale base for a more interventive approach in favor of a surgical resection of oligometastatic disease.

Our results support the evidence that surgical indication confirms its important role in stage IV melanoma with resectable pulmonary metastases, and selected patients can still benefit from pulmonary metastasectomy in terms of overall cancer-specific survival. Furthermore, the novel systemic therapies may contribute to prolonged survival after systemic recurrence following pulmonary metastasectomy.

Patients with long DFI, radial growth melanoma phase and no site of metastatization other than lung seem to be the best candidates for lung metastasectomy.

However, to drive stronger conclusions, further studies evaluating the role of metastasectomy in patients with PmMM are needed.

## Figures and Tables

**Table 1:** Patients’ clinicopathological characteristics.

Variables	#183 Patients
Sex (male)	76 (41.5%)
Previous oncological history	21 (11.4%)
Melanoma primary site:	
Skin	172 (94.0%)
Uvea	9 (4.9%)
Mucosae	2 (1.1%)
Breslow thickness (mm)	3.11 ± 3.26
Histology	
Acral	19 (10.4%)
Superficial spreading	95 (51.9%)
Nodular	65 (35.5%)
Lentigo	4 (2.2%)
B-RAF mutation	64 (34.9%)
Stage at diagnosis	10 (5.5%)
IA	36 (19.7%)
IB	48 (26.2%)
IIA	22 (12.0%)
IIB	14 (7.7%)
IIC	20 (10.9%)
IIIA	13 (7.1%)
IIIB	4 (2.2%)
IIIC	1 (0.5%)
IIID	15 (8.2%)
IV	
Type of adjuvant therapy:	
None	87 (47.6%)
Immunotherapy	75 (41.0%)
Targeted Therapy	18 (9.8%)
Chemotherapy	3 (1.6%)
Patients with previous metastases other than lung	24 (13.1%)
Diameter of pulmonary metastases (mm)	12.4 ± 5.6
Multiple lung metastases	21 (11.5%)
Synchronous metastases	26 (14.2%)
Type of lung resection:	
Wedge	175 (95.6%)
Segmentectomy/Lobectomy	8 (4.4%)
Minor complications after metastasectomy	21 (11.5%)
Major complications after metastasectomy	null
In-hospital stay (days)	4.46 ± 2.8
Thirty-day mortality	null
Adjuvant therapy after metastasectomy:	
None	19 (10.4%)
Immunotherapy	86 (47.0%)
Targeted therapy	78 (42.6%)
Recurrence of disease after metastasectomy	73 (39.9%)

**Table 2:** Univariate and multivariable analyses for prognostic factors on cancer specific survival from melanoma resection.

Variables	Univariate Analysis	Multivariable Analysis	
	*p*-Value	HR [95% CI]	*p*-Value
Previous oncological history	0.370		
Melanoma histology	**0.052**	1.295 [0.699–2.398]	0.411
Vertical growth	0.122		
Radial growth	0.524		
Breslow thickness	0.636		
BRAF mutation	0.103		
Neurotropism	0.858		
Vascular invasion	0.999		
Multiple lung metastases	**0.002**	1.508 [0.540–4.206]	0.433
Synchronous lung metastasis	**<0.001**	1.130 [0.326–3.923]	0.847
Previous metastatic sites other than lung	**0.0002**	1.607 [0.443–5.834]	0.471
Diameter of metastasis > 2 cm	0.547		
Type of lung resection	0.986		
Therapy after metastasectomy	0.185		
DFI < 24 months	**0.002**	7.813 [2.787–21.902]	**<0.001**

**Table 3:** Univariate and multivariable analyses for prognostic factors on cancer specific survival from metastasectomy.

Variables	Univariate Analysis	Multivariable Analysis	
	*p*-Value	HR [95% CI]	*p*-Value
Previous oncological history	0.883		
Melanoma histology	0.069		
Vertical growth	**0.018**	**9.688 [1.410–66.551]**	**0.021**
Radial growth	0.784		
Breslow thickness	0.320		
BRAF mutation	0.067		
Neurotropism	0.854		
Vascular invasion	0.680		
Multiple lung metastases	0.054	2.687 [0.614-11.753]	0.189
Synchronous lung metastasis	0.146		
Previous metastatic sites other than lung	**<0.001**	**3.154 [1.332–7.470]**	**0.009**
Diameter of metastasis > 2 cm	0.592		
Type of lung resection	0.902		
Therapy after metastasectomy	0.435		
DFI < 24 months	**0.007**	1.670 [0.267–10.456]	0.584

**Table 4:** Univariate and multivariable analyses for prognostic factors on DFI between primary melanoma and first pulmonary metastasis diagnosis.

Variables	Univariate Analysis	Multivariable Analysis	
	*p*-Value	HR [95% CI]	*p*-Value
Male sex	0.914		
Melanoma histology	0.143		
Vertical growth	0.182		
Breslow thickness	0.636		
BRAF mutation	0.029	1.285 [0.894–1.846]	0.176
c-KIT mutation	0.429		

**Table 5:** Univariate and multivariable analyses for prognostic factors on disease-free survival after pulmonary metastasectomy and before disease recurrence.

Variables	Univariate Analysis	Multivariable Analysis	
	*p*-Value	HR [95% CI]	*p*-Value
Male sex	0.298		
Melanoma histology	0.099		
Vertical growth	0.616		
BRAF mutation	0.131		
c-KIT mutation	0.685		
Breslow thickness	0.407		
Synchronous lung metastasis	0.484		
Diameter of metastasis > 2 cm	0.848		
Type of lung resection	0.241		
Therapy after metastasectomy	**<0.001**	0.106 [0.028–0.404]	**0.001**

## Data Availability

The data presented in this study are available in this article and can be shared up on request.

## References

[1] Balch C.M., Gershenwald J.E., Soong S.J., Thompson J.F., Atkins M.B., Byrd D.R., Buzaid A.C., Cochran A.J., Coit D.G., Ding S. (2009). Final version of 2009 AJCC melanoma staging and classification. J. Clin. Oncol..

[2] Siegel R.L., Miller K.D., Jemal A. (2020). Cancer statistics, 2020. CA Cancer J. Clin..

[3] Atkins M.B., Hsu J., Lee S., Cohen G.I., Flaherty L.E., Sosman J.A., Sondak V.K., Kirkwood J.M. (2008). Phase III trial comparing concurrent biochemotherapy with cisplatin, vinblastine, dacarbazine, interleukin-2, and interferon alfa-2b with cisplatin, vinblastine, and dacarbazine alone in patients with metastatic malignant melanoma (E3695): A trial coordinated by the Eastern Cooperative Oncology Group. J. Clin. Oncol..

[4] Viehofa J., Livingstone E., Loschaa E., Stockhammera P., Bankfalvid A., Plo¨nesa T., Mardanzaia K., Zimmerb L., Suckerb A., Schadendorfb D. (2019). Prognostic factors for pulmonary metastasectomy in malignant melanoma: Size matters. Eur. J. Cardiothorac. Surg..

[5] Hanna T.P., Chauvin C., Miao Q., Rizkalla M., Ried K., Peng Y., Nguyen P., Jalink D., An Nanki S. (2018). Clinical Outcomes After Pulmonary Metastasectomy for Melanoma: A Population-Based Study. Ann. Thorac. Surg..

[6] Bhatia S., Tykodi S.S., Thompson J.A. (2009). Treatment of metastatic melanoma: An overview. Oncology.

[7] Passarelli A., Mannavola F., Stucci L.S., Tucci M., Silvestris F. (2017). Immune system and melanoma biology: A balance between immune surveillance and immune escape. Oncotarget.

[8] Deutsch G.B., Flaherty D.C., Kirchoff D.D., Bailey M., Vitug S., Foshag L.J., Fraies M.B., Bilchick A.J. (2017). Association of Surgical Treatment, Systemic Therapy, and Survival in Patients with Abdominal Visceral Melanoma Metastases, 1965–2014: Relevance of Surgical Cure in the Era of Modern Systemic Therapy. JAMA Surg..

[9] Meacci E., Nachira D., Zanfrini E., Evangelista J., Triumbari E.K.A., Congedo M.T., Petracca Ciavarella L., Chiappetta M., Vita M.L., Schinzari G. (2021). Prognostic Factors Affecting Survival after Pulmonary Resection of Metastatic Renal Cell Carcinoma: A Multicenter Experience. Cancers.

[10] Oliaro A., Filosso P.L., Bruna M.C., Mossetti C., Ruffini E. (2010). Pulmonary metastasectomy for melanoma. J. Thorac. Oncol..

[11] Wasif N., Bagaria S.P., Ray P., Morton D.L. (2011). Does metastasectomy improve survival in patients with Stage IV melanoma? A cancer registry analysis of outcomes. J. Surg. Oncol..

[12] Sosman J.A., Moon J., Tuthill R.J., Warneke J.A., Vetto T.J., Redman B.G., Liu P.Y., Unger J.M., Flaherty L.E., Sondak V.K. (2011). A phase 2 trial of complete resection for stage IV melanoma: Results of Southwest Oncology Group Clinical Trial S9430. Cancer.

[13] Harrison Howard J., Thompson J.F., Mozzillo N., Nieweg O.E., Howkstra H.J., Roses D.F., Sondak V.K., Reintgen D.S., Kashani-Sabet M., Karakousis C.P. (2012). Metastasectomy for distant metastatic melanoma: Analysis of data from the first Multicenter Selective Lymphadenectomy Trial (MSLT-I). Ann. Surg. Oncol..

[14] Schuhan C., Muley T., Dienemann H., Pfannschmidt J. (2011). Survival after pulmonary metastasectomy in patients with malignant melanoma. Thorac. Cardiovasc. Surg..

[15] Van Akkooj A.C.J., Atkins M.B., Agarwala S.S., Lorigan P. (2016). Surgical Management and Adjuvant Therapy for High-Risk and Metastatic Melanoma. Am. Soc. Clin. Oncol. Educ. Book.

[16] Hodi F.S., O’Day S.J., McDermott D.F., Weber R.W., Sosman J.A., Haanen J.B., Gonzalez R., Robert C., Schadendorf D., Hassel J.C. (2010). Improved survival with ipilimumab in patients with metastatic melanoma. N. Engl. J. Med..

[17] Chapman P.B., Hauschild A., Robert C., Haanen J.B., Ascierto P., Larkin J., Dummer R., Garbe C., Testori A., Maio M. (2011). Improved survival with vemurafenib in melanoma with BRAF V600E mutation. N. Engl. J. Med..

[18] Wolchok J.D., Chiarion-Sileni V., Gonzalez R., Rutkowski P., Jrob J.J., Cowey L., Lao C.D., Wagstaff J., Schadendorf D., Ferrucci P.F. (2017). Overall Survival with Combined Nivolumab and Ipilimumab in Advanced Melanoma. N. Engl. J. Med..

[19] Hodj F.S., Chiarion-Sileni V., Gonzalez R., Grob J.J., Rutkowski P., Cowey C.L., Lao C.D., Schadendorf D., Wagstaff D., Dummer R. (2018). Nivolumab plus ipilimumab or nivolumab alone versus ipilimumab alone in advanced melanoma (CheckMate 067): 4-year outcomes of a multicentre, randomised, phase 3 trial. Lancet Oncol..

[20] Michielin O., van Akkooi A.C.J., Ascierto P.A., Dummer R., on behalf of the ESMO Guidelines Committee (2019). Cutaneous melanoma: ESMO Clinical Practice Guidelines for diagnosis, treatment and follow-up. Ann. Oncol..

[21] Faries M.B., Mozzillo N., Kashani-Sabet M., Thompson J.F., Kelley M.C., DeConti R.C., Lee J.E., Huth J.F., Wagner J., Dalgleish A. (2017). Long-Term Survival after Complete Surgical Resection and Adjuvant Immunotherapy for Distant Melanoma Metastases. Ann. Surg. Oncol..

[22] Leo F., Cagini L., Rocmans P., Cappello M., Geel A.N., Maggi G., Goldstraw P., Pastorino U. (2000). Lung metastases from melanoma: When is surgical treatment warranted?. British J. Cancer.

[23] Meyer T., Merkel S., Goehl J., Hohenberger W. (2000). Surgical therapy for distant metastases of malignant melanoma. Cancer.

[24] Feun L.G., Gutterman J., Burgess M.A., Hersh E.M., Mavligit G., McBride C.M., Benjamin R.S., Richman S.P., Murphy W.K., Bodey G.P. (1982). The natural history of resectable metastatic melanoma (Stage IVA melanoma). Cancer.

[25] Overett T.K., Shiu M.H. (1985). Surgical treatment of distant metastatic melanoma. Indications and results. Cancer.

[26] Wong J.H., Euhus D.M., Morton D.L. (1988). Surgical resection for metastatic melanoma to the lung. Arch. Surg..

[27] Karp N.S., Boyd A., DePan H.J., Harris M.N., Ros D.F. (1990). Thoracotomy for metastatic malignant melanoma of the lung. Surgery.

[28] Gorenstein L.A., Putnam J.B., Nataraian G., Balch C.A., Roth J.A. (1991). Improved survival after resection of pulmonary metastases from malignant melanoma. Ann. Thorac. Surg..

[29] Harpole D.H., Johnson C.M., Wolfe W.G., George S.L., Seigler H.F. (1992). Analysis of 945 cases of pulmonary metastatic melanoma. J. Thorac. Cardiovasc. Surg..

[30] Wankhede D., Grover S. (2022). Outcomes After Curative Metastasectomy for Patients with Malignant Melanoma: A Systematic Review and Metaanalysis. Ann. Surg. Oncol. Jan..

[31] Pastorino U., Buyse M., Friedel G., Ginsberg R.J., Girard P., Goldstraw P., Johnston M., McCormack P., Pass H., Putnam Jr J.B. (1997). Long-term results of lung metastasectomy: Prognostic analyses based on 5206 cases. J. Thorac. Cardiovasc. Surg..

[32] Dalrymple-Hay M.J., Rome P.D., Kennedy C., Fulham M., McCaughan B.C. (2002). Pulmonary metastatic melanoma—the survival benefit associated with positron emission tomography scanning. Eur. J. Cardiothorac. Surg..

[33] Andrews S., Robinson L., Cantor A., De Conti R.C. (2006). Survival after surgical resection of isolated pulmonary metastases from malignant melanoma. Cancer Control.

[34] Neuman H.B., Patel A., Hanlon C., Wolchok J.D., Houghton A.N., Coit D.G. (2007). Stage-IV melanoma and pulmonary metastases: Factors predictive of survival. Ann Surg. Oncol..

[35] Petersen R.P., Hanish S.I., Haney J.C., Miller C.C., Burfeind W.R., Tyler D.S., Seiger H.F., Wolfe W., D’Amico T.A., Harpole D.H. (2007). Improved survival with pulmonary metastasectomy: An analysis of 1720 patients with pulmonary metastatic melanoma. J. Thorac. Cardiovasc. Surg..

[36] Chua T.C., Scolver R.A., Kennedy C.W., Yan T.D., McCaughan B.C., Thompson J.F. (2012). Surgical management of melanoma lung metastasis: An analysis of survival outcomes in 292 consecutive patients. Ann. Surg. Oncol..

[37] Smith M.J., Smith H.G., Joshi K., Gore M., Strauss D.C., Hayes A.J., Larkin J. (2018). The impact of effective systemic therapies on surgery for stage IV melanoma. Eur. J. Cancer.

[38] Bello D.M., Panageas K.S., Hollmann T., Shoushtari A.N., Momtaz P., Chapman P.B., Postow M.A., Callahan M.K., Wolchock J.D., Brady M.S. (2020). Survival outcomes after metastasectomy in melanoma patients categorized by response to checkpoint blockade. Ann. Surg. Oncol..

[39] Day C.L., Lew R.A., Mihm M.C., Sober A.J., Harris M.N., Kopf A.W., Fitzpatrick T.B., Harrist T.J., Golomb F.M., Postel A. (1982). A multivariate analysis of prognostic factors for melanoma patients with lesions greater than or equal to 3.65 mm in thickness. The importance of revealing alternate Cox models. Ann. Surg..

[40] Day C.L., Mihm M.C., Sober A.J., Harris M.N., Kopf A.W., Fitzpatrik T.B., Lew R.A., Harrist T.J., Golomb F.M., Postel A. (1982). Prognostic factors for melanoma patients with lesions 0.76–1.69 mm in thickness. An appraisal of ‘thin’ level IV lesions. Ann. Surg..

[41] Day C.L., Mihm M.C., Lew R.A., Harris M.N., Kopf A.W., Fitzpatrick T.B., Harrist T.J., Golomb F.M., Postel A., Hennessey P. (1982). Prognostic factors for clinical stage I melanoma of intermediate thickness (1.51–3.39 mm). A conceptual model for tumor growth and metastasis. Ann. Surg..

[42] Crowson A.N., Magro C., Mihm M.C., Hearing V.J., Leong S.P.L. (2006). The biology of melanoma progression: From melanocyte to metastatic seed. From Melanocytes to Malignant Melanoma.

[43] Bedrosian I., Faries M.B., Guerry D., Elenitsas R., Schuchter L., Mick R., Spitz F.R., Bucky L.P., Alavi A., Elder D.E. (2000). Incidence of sentinel node metastasis in patients with thin primary melanoma (£1 mm) with vertical growth phase. Ann. Surg. Oncol..

[44] Green A.C., Williams G.M., Logan V., Strutton G.M. (2011). Reduced melanoma after regular sunscreen use: Randomized trial follow-up. J Clin. Oncol..

[45] Boniol M., Autier P., Boyle P., Gandini S. (2012). Cutaneous melanoma attribuible to sunbeds use: Systematic review and metanalysis. BMJ.

[46] Aberg T. (1997). Selection mechanisms as major determinants of survival after pulmonary metastasectomy. Ann. Thorac. Surg..

[47] Aberg T., Malmberg K.A., Nilsson B., Nou E. (1980). The effect of metastasectomy: Fact or fiction?. Ann. Thorac. Surg..

[48] Treasure T., Milosevic M., Fiorentino F., Macbeth F. (2014). Pulmonary metastasectomy: What is the practice and where is the evidence for effectiveness?. Thorax.

